# Information Needs Priorities in Patients Diagnosed With Cancer: A Systematic Review

**Published:** 2014-03-01

**Authors:** Joseph D. Tariman, Ardith Doorenbos, Karen G. Schepp, Seema Singhal, Donna L. Berry

**Affiliations:** From ^1^School of Nursing/College of Science and Health at De Paul University, Chicago, Illinois; ^2^University of Washington, Seattle, Washington; and ^3^Dana Farber Cancer Institute, Boston, Massachusetts

## Abstract

Information-sharing is an integral part of cancer care. Several studies have examined the information needs of patients with various types of cancer. However, the priorities of information needs among patients with cancer have not been reported. A systematic review was performed to identify published studies that examined priorities of information needs in patients with cancer. PubMed (1966 to February 2012), PsycINFO (1967 to February 2012), and CINAHL (1982 to February 2012) databases were searched to access relevant medical, psychological, and nursing literature. Thirty studies involving patients with breast, prostate, lung, colorectal, gynecologic, hematologic, and other cancers revealed patients’ information needs priorities. The top three patient information priorities were related to prognosis, diagnosis, and treatment options. The top information priorities reported in this systematic review could serve as a start to elicit patients’ information needs and guide patient education across the cancer care continuum. Being able to prioritize the most-needed information can make patient encounters more meaningful and useful.

Research studies on information needs and information-sharing in patients with cancer have been increasing steadily in the past 3 decades. These studies relating to patient information-sharing were aimed at improving patient education and ultimately increasing patient participation in health-care decision-making (Chouliara, Kearney, Stott, Molassiotis, & Miller, 2004; Gaston & Mitchell, 2005; Husson, Mols, & van de Poll-Franse, 2010; Rutten, Arora, Bakos, Aziz, & Rowland, 2005). Two systematic reviews on information-sharing in patients with advanced cancer revealed that patients with cancer indeed have unmet information needs (Gaston & Mitchell, 2005; Rutten et al., 2005). Moreover, a systematic review of the priorities of patients with cancer with respect to their information needs has not been previously reported.

Providing health-care information to patients, caregivers, and family members is considered an important aspect of cancer care (Jacobson et al., 2009). In a seminal paper, Degner and colleagues (1998) argued that in an era of scarce health-care resources, patient information needs are best prioritized. Prioritization directs the attention of clinicians to the most important information needs, enhances the delivery of information that patients need, and provides relevant information to patients at specific periods of their illness. Additionally, prioritization of information needs can make patient encounters more relevant to the patients’ actual or perceived needs (Degner et al., 1997; 1998).

Obtaining information, particularly regarding prognosis and treatment, remains a major area of need for individuals with cancer (Nagler et al., 2010; Rutten et al., 2005). Evidence shows that most patients with cancer want to participate in the decision-making process (Tariman, Berry, Cochrane, Doorenbos, & Schepp, 2010). In order to truly help patients make autonomous decisions, oncology clinicians must provide accurate, timely, and meaningful information. Because resources are limited, prioritizing patients’ information needs is an important step toward efficiency.

Researchers in the field of information-sharing postulate that prioritization of the patient’s information needs potentially offers the following advantages (Bilodeau & Degner, 1996; Degner et al., 1997; 1998):

- Directs the attention of oncology clinicians to the highest information needs
- Guides oncology clinicians to prioritize patient teaching and information sharing
- Saves time and enhances the quality of information that patients will receive
- Provides relevant information to patients at specific points on their disease and re-covery trajectory
- Makes clinician-patient encounters more meaningful and on target
- Lowers the psychological distress associated with treatment decision-making
- Helps patients assume a more active role in decision-making

The purpose of this review article is to summarize relevant studies that have examined information needs priorities in patients with various types of cancers, identifying the prioritized information needs across the studies. Moreover, this review also summarizes the association of age with patients’ priorities of information needs and describes the trend over time. The implications of the findings for practice and research are also discussed. 

## Methods

A systematic review of the research literature was conducted to identify studies that examined the information needs priorities in patients diagnosed with cancer. The PubMed (1966 to February 2012), PsycINFO (1986 to February 2012), and CINAHL (1982 to February 2012) databases were searched to access relevant medical, psychological, and nursing literature. The medical subject heading terms that were individually or simultaneously used during the search were cancer, information needs, patient education, and patient participation. The search was limited to research articles concerning adults, written in English, and published in peer-reviewed journals.

A total of 136 articles were initially retrieved; abstracts were individually reviewed for any mention of information needs priorities. If information needs priorities were reported, full-text copies of the articles were then reviewed in depth. Of the 136 articles, 37 full-text articles were retrieved and reviewed, yielding 30 studies reporting information needs priorities in patients with various types of cancer.

The information needs priorities from published studies were entered into Predictive Analytic SoftWare (PASW) Statistics version 18 (SPSS, 2009). The top three priorities were calculated using simple frequencies and percentages and tabulated according to rank.

## Results

Table 1 outlines a summary of 30 studies regarding the information needs priorities of patients with various cancers. These studies are grouped by type of cancer diagnosis. Overall, the top three information needs priorities among cancer patients are information related to prognosis or likelihood of cure, disease stage, and treatment options (Table 2).

**Table 1:**
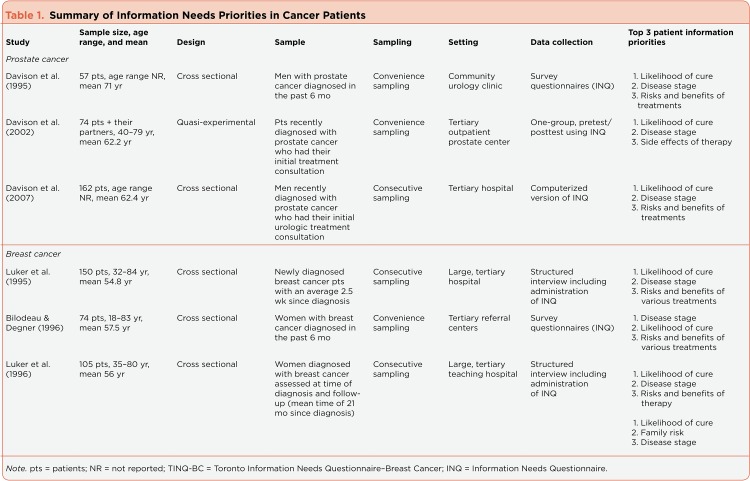
Table 1. Summary of Information Needs Priorities in Cancer Patients

**Table 1:**
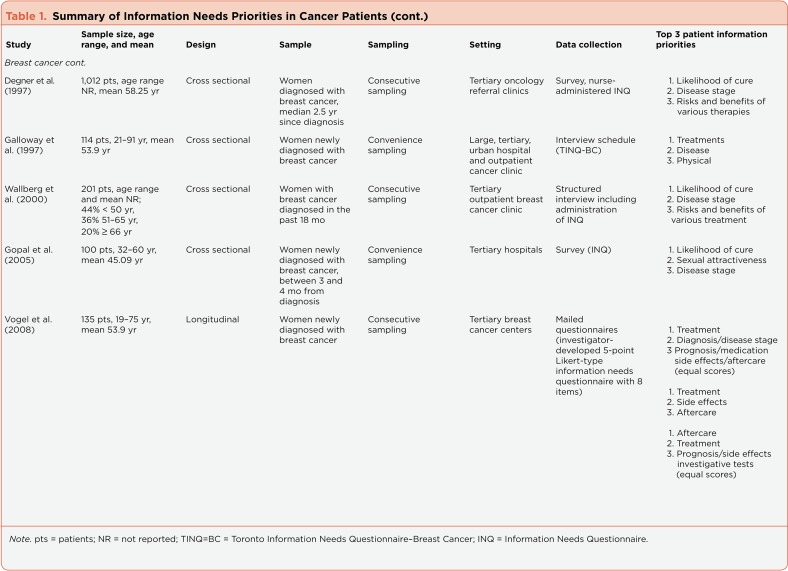
Table 1. Summary of Information Needs Priorities in Cancer Patients

**Table 1:**
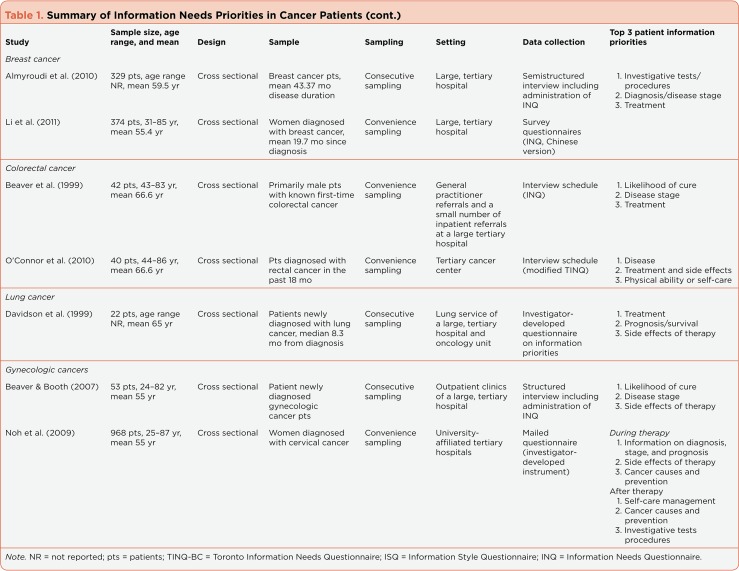
Table 1. Summary of Information Needs Priorities in Cancer Patients

**Table 1:**
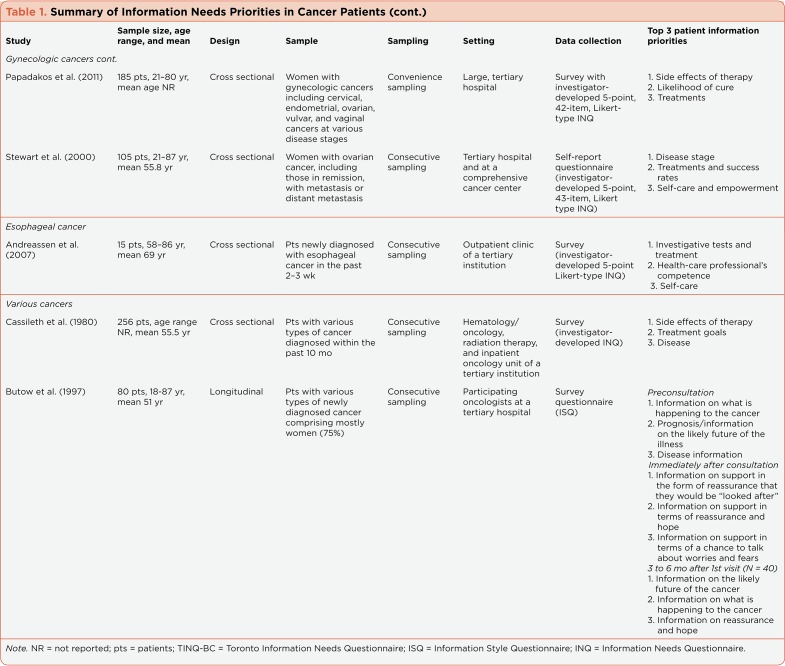
Table 1. Summary of Information Needs Priorities in Cancer Patients

**Table 1:**
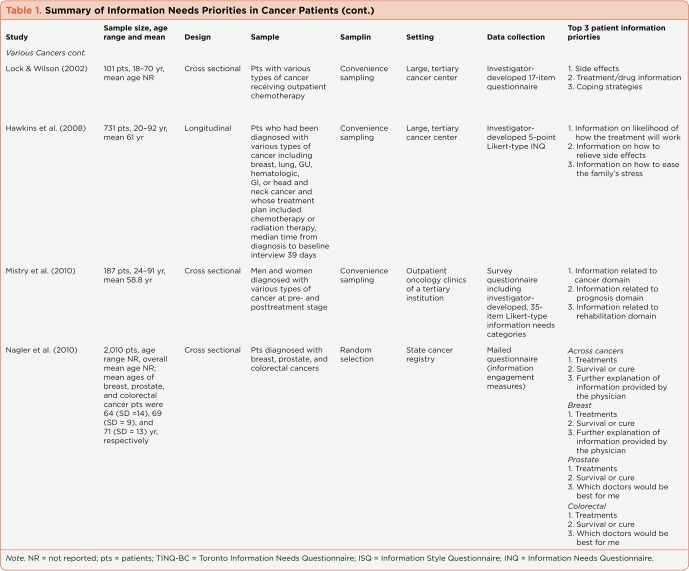
Table 1. Summary of Information Needs Priorities in Cancer Patients

**Table 1:**
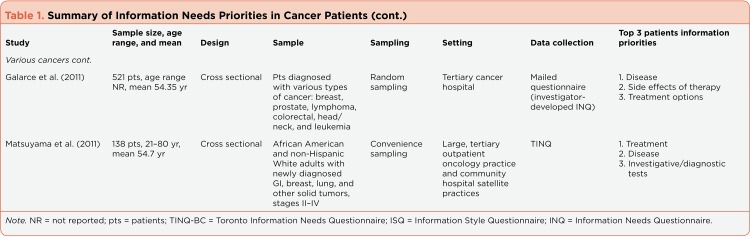
Table 1. Summary of Information Needs Priorities in Cancer Patients

**Table 2:**
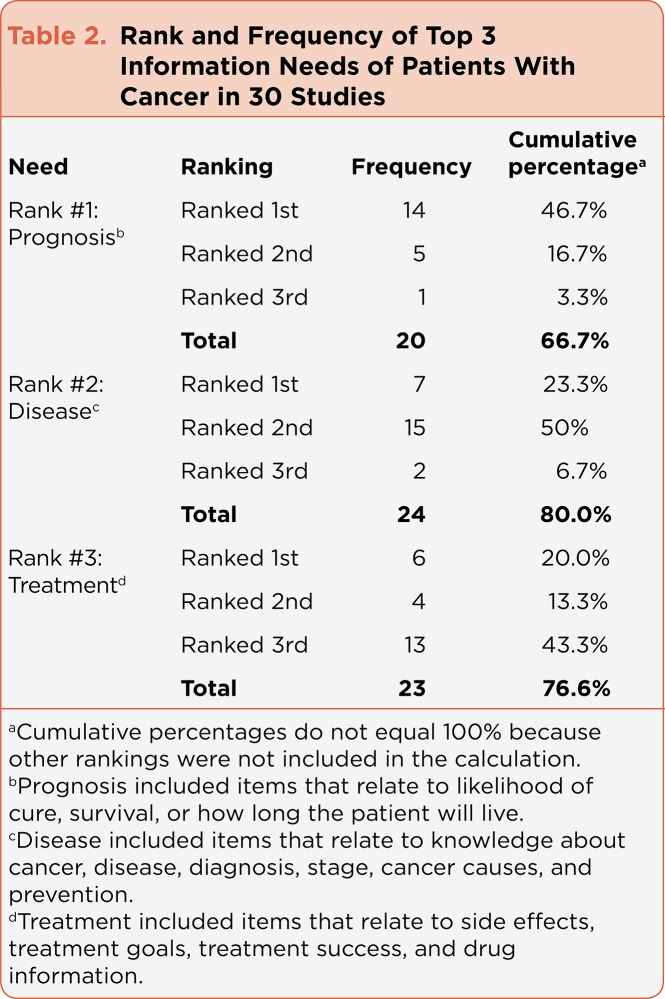
Table 2. Rank and Frequency of Top 3 Information Needs of Patients With Cancer in 30 Studies

The majority of studies have been conducted in women with breast cancer and in individuals with various types of cancers. A few other studies were conducted in patients with gynecological, prostate, colorectal, esophageal, and lung cancers. Only 4 out of 30 studies have been done longitudinally, which could be attributed to the limited time and resources clinicians and researchers have to assess patients’ priorities of information needs at scheduled intervals. The most commonly used tools to assess information priorities were the Information Needs Questionnaire (Degner el., 1998) and various investigator-developed questionnaires.

## Impact of Age on Information Needs Priorities

Age has been examined for an association with patients’ information needs priorities. Two studies (Degner et al., 1997; Wallberg et al., 2000) reported that information about sexuality and physical attractiveness was more important to younger women (< 50 years) than older women ( 50 years; *p* < .001). Luker and colleagues (1995) also reported similar findings. Similarly, Davison and colleagues (2002) reported that young men (< 65 years) ranked information on sexuality as more important than older men ( 65 years). However, older women ( 60 years) rated information pertaining to social life as more important than did younger women (*p* = .03; Luker et al., 1995). Bilodeau and Degner (1996) reported age (specifically within the 65- to 85-year-old category) to be significantly associated with a higher ranking for self-care information (p .02).

## Discussion

This systematic review reveals that decision researchers consistently find that there is a discernible priority of information needs among cancer patients, needs that include prognosis, diagnosis, treatments, and side effects. Although clear patterns exist in this research, variations in patients’ reported information needs priorities remain across the different types of cancer. Moreover, some longitudinal studies included in this review have suggested that preferences and priorities do change over time for individuals. However, it is unclear whether these changes are influenced by the type of cancer diagnosis, the stage of the disease, and/or the age of individuals. More longitudinal studies are needed to better understand the different factors that may affect information priorities over time.

Overall, the top three information priorities included prognosis, disease, and treatments. These priorities are not surprising, as cancer remains a devastating disease. Most cancers are largely incurable except when they are diagnosed at an early stage (e.g., breast and prostate cancers), and some are aggressive and fatal. We (the authors) postulate that the patients would want to know first how long they are going to live in order to prepare for the inevitable. However, none of the studies included in this review provided additional information as to why patients chose prognosis first among other information needs priorities. Understanding the disease and related cancer treatments were second and third priorities, respectively. It is natural for patients to want to know what type of cancer and what stage of the disease they have, as most patients fear certain types of cancer and advanced stage of the disease since they are usually associated with shorter survival. With the advent of many novel cancer agents, it is expected that patients would want to know about the different treatments available to them. A growing number of cancer patients prefer to participate in making treatment decisions; knowing about the different treatment options is the first step toward active patient participation (Tariman et al., 2010). 

This review revealed that age could influence patients’ information needs in terms of prioritizing sexual attractiveness in younger patients and self-care in older adult patients. There is a common belief that the younger the patient, the more likely he or she is to put more importance on sexuality and the impact of cancer on sexual relationships. Since most studies included in this review were conducted in Europe and North America, it also is not surprising to find that older adults wanted to know more about self-care, reflecting autonomy as one of the top values among westerners (Martin & Roberto, 2006).

It is important that health-care clinicians assess their patients’ individual information needs priorities. Large longitudinal studies involving well-diversified patient populations with various types of cancer are needed to validate the top information priorities reported in this review. As we are now living in the digital era, innovative educational intervention studies using the Internet and computer aids are needed to meet patients’ information needs priorities and improve efficiency in delivering that information.

## Limitations

Despite the number of reviewed studies that examined information needs priorities in patients with cancer, the authors recognized certain limitations related to missing data. First, unpublished dissertation studies were not included in the search. Second, other databases such as Google Scholar or Web of Science were not in cluded in the search. Finally, only studies written in the English language and conducted in North America and Europe were included in this review, limiting generalizability to non–English-speaking countries. Finally, most of the studies included were conducted in a tertiary care facility, limiting the generalizability of the findings to other care settings such as private, community-based clinics.

## Implications for Advanced Practitioners

Advanced oncology practitioners, who are generally responsible for providing patient education, can use the top three priorities of prognosis, stage of disease, and treatment options reported in this review as a starting place when assessing their own patients’ information needs. Focusing on the information that each patient considers to be a priority could potentially lead to better cancer care.

Advanced practice professionals must be aware that different socioeconomic and cultural factors can have the potential to influence a patient’s information needs. A better understanding of the various influences on information priorities could help in meeting the needs of patients in general and in individual cases.

## Conclusions

Patients with various types of cancer have information needs priorities. These priorities most commonly include information that relates to prognosis, disease, and treatments. Age could play an important factor in information needs priorities. Younger patients tend to put more importance on information related to sexuality, while older adults prioritize information related to self-care. Future research should consider examining how age (young adult, adult, and the elderly) and gender influence priority of information needs in cancer patients. Prospective, longitudinal studies that examine the factors that influence information needs priorities over time are needed. Interventional studies geared toward improving efficiency in delivering patient information are also needed.

## Acknowledgments

J.D.T. contributed to the extensive literature review and manuscript drafting. A.D., K.G.S., S.S., and D.L.B. participated in manuscript design and critique of the paper. All authors have read and approved the final manuscript. The authors would like to thank Barbara Cochrane, PhD, for her initial review of this paper. 

## Funding

This work was supported by the National Institutes of Health (NR07106, F31NR011124) and the Achievement Rewards for College Scientists (ARCS) Foundation, Seattle (pre-doctoral fellowship, 2006-2009). The content is solely the responsibility of the authors and does not necessarily represent the official views of the NIH and the ARCS Foundation.
